# Heart Failure Management through Telehealth: Expanding Care and Connecting Hearts

**DOI:** 10.3390/jcm13092592

**Published:** 2024-04-28

**Authors:** Andrea Tedeschi, Matteo Palazzini, Giancarlo Trimarchi, Nicolina Conti, Francesco Di Spigno, Piero Gentile, Luciana D’Angelo, Andrea Garascia, Enrico Ammirati, Nuccia Morici, Daniela Aschieri

**Affiliations:** 1 Cardiology Unit of Emergency Department, Guglielmo da Saliceto Hospital, 29121 Piacenza, Italy; francesco.dispigno@yahoo.com (F.D.S.); d.aschieri@ausl.pc.it (D.A.); 2 “De Gasperis” Cardio Center, Niguarda Hospital, ASST Grande Ospedale Metropolitano Niguarda, 20162 Milan, Italy; matteo.palazzini@ospedaleniguarda.it (M.P.); nicolina.conti@ospedaleniguarda.it (N.C.); piero.gentile@ospedaleniguarda.it (P.G.); luciana.dangelo@ospedaleniguarda.it (L.D.); andrea.garascia@ospedaleniguarda.it (A.G.); enrico.ammirati@ospedaleniguarda.it (E.A.); 3 Department of Clinical and Experimental Medicine, University of Messina, 98100 Messina, Italy; giancarlo.trimarchi18@gmail.com; 4 IRCCS Fondazione Don Carlo Gnocchi, 20148 Milan, Italy; nmorici@dongnocchi.it

**Keywords:** telemedicine, heart failure, implementation, connection, artificial intelligence

## Abstract

Heart failure (HF) is a leading cause of morbidity worldwide, imposing a significant burden on deaths, hospitalizations, and health costs. Anticipating patients’ deterioration is a cornerstone of HF treatment: preventing congestion and end organ damage while titrating HF therapies is the aim of the majority of clinical trials. Anyway, real-life medicine struggles with resource optimization, often reducing the chances of providing a patient-tailored follow-up. Telehealth holds the potential to drive substantial qualitative improvement in clinical practice through the development of patient-centered care, facilitating resource optimization, leading to decreased outpatient visits, hospitalizations, and lengths of hospital stays. Different technologies are rising to offer the best possible care to many subsets of patients, facing any stage of HF, and challenging extreme scenarios such as heart transplantation and ventricular assist devices. This article aims to thoroughly examine the potential advantages and obstacles presented by both existing and emerging telehealth technologies, including artificial intelligence.

## 1. Introduction

Telemedicine—or telehealth—the practice of delivering healthcare remotely using telecommunications technologies, has significantly influenced cardiology, improving patient care and outcomes by harnessing technology to enhance remote monitoring (RM), diagnostics, and communication between healthcare providers [1,2,3].

Heart failure (HF) affects a significant portion of the adult population in developed countries, imposing a substantial economic burden on healthcare systems due to its high morbidity and mortality rate [4]. In striving to improve the management of patients affected by this challenging condition, spurred by the wave of post-pandemic healthcare renewal, telemedicine has emerged as an increasingly utilized tool, serving as a versatile resource applicable in all phases of this complex disease [3].

The integration of various implements, ranging from basic devices for monitoring body weight, blood pressure, and oxygen saturation, to wearable technology and state-of-the-art RM systems incorporated into Cardiovascular Implantable Electronic Devices (CIEDs), plays a well-established role in managing HF patients [3]. Although RM in HF has traditionally been focused on lifestyle management [5], early recognition of HF exacerbation signs, and subsequent diuretic adjustment [6,7,8], the pursuit of optimizing medical therapy could represent a new significant frontier. Alongside these advancements, the role of invasive hemodynamic monitoring devices is increasingly emerging, proving effective in reducing both hospitalizations for HF and positively impacting patients’ mortality rates [9,10]. Their utilization especially holds great promise, particularly in enhancing the management of patients with advanced heart failure (AHF) and those with left ventricular assist devices (LVADs) [11]. Additionally, telehealth is gaining ground as a crucial resource in the care of patients undergoing heart transplantation (HTx), serving not only for clinical monitoring but also for educational and rehabilitative purposes [12,13].

As further evidence of its versatility, telehealth has proven effective not only in the field of HF but also in patients with cardiac electrophysiology diseases [14].

Despite the undeniable benefits offered by telemedicine in managing HF patients, there are some limitations, particularly in terms of the lack of equal accessibility for the most vulnerable patients [3]. Health networks and clinicians should strive to incorporate telehealth into HF management while addressing concerns about access and infrastructure. Investment in technologies and connectivity is crucial to ensure that telehealth does not widen health disparities [15]. In this context, the utilization of artificial intelligence (AI) undoubtedly represents one of the most compelling and forward-thinking perspectives in advancing towards accessible and effective telemedicine [16].

This article presents a comprehensive narrative review exploring telehealth in the context of HF, drawing insights from a wide range of sources including the medical and scientific literature, as well as Internet-based research on telemedicine projects and studies. The central focus of this review is a detailed examination of the telehealth landscape, particularly its potential benefits and challenges in addressing HF. The research is specifically aimed at identifying and discussing the primary methods of telemedicine application within HF treatment, such as telehealth consultations, the use of wearable devices, the integration of implantable cardiac devices, and the advancement of remote invasive hemodynamic monitoring techniques. Through this thorough analysis, this article seeks to underscore the significant impact that current and emerging telehealth technologies are having on the management and treatment of HF, providing a critical perspective on their roles in improving patient care and tackling the complexities associated with this chronic condition.

**Figure 1:**
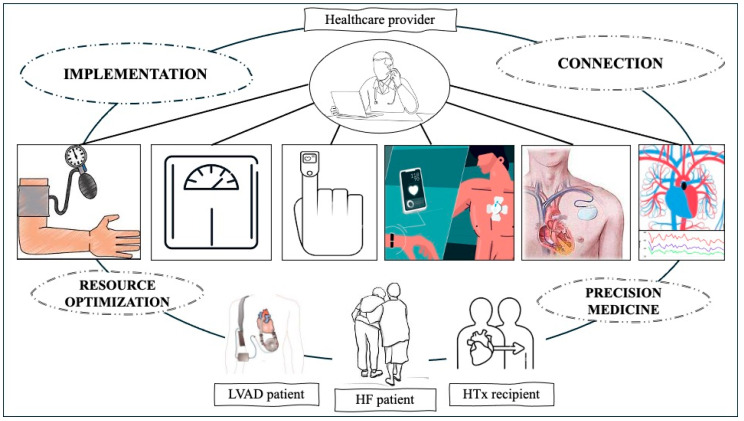
Telemedicine as a tool to achieve implementation, resource optimization, connection, and progress towards precision medicine. HF: heart failure; HTx: heart transplantation; LVAD: left ventricular assist device.

By doing so, it seeks to contribute to the ongoing dialogue surrounding the optimization of patient care and outcomes across the different stages of HF, within the context of its evolutionary natural history (Figure 1).

## 2. A Brief Chronicle of Telemedicine in Heart Failure: In the Drama of the Pandemic, the Turn towards the Future

Telehealth in cardiology has a rich history, dating back to the mid-20th century with the utilization of technologies such as Holter monitors and implantable loop recorders. Moreover, emergency medical services have embraced telemedicine, facilitating the swift transmission of vital information like electrocardiograms from the field to emergency departments, resulting in expedited responses for conditions such as acute ST-elevation myocardial infarctions [17]. Among the various applications of telemedicine, HF has emerged as a prominent field where its utilization is well established. Although initial experiences, primarily focused on the multiparameter home monitoring of frail HF patients, were documented as early as the early 2000s [18], the COVID-19 pandemic unequivocally solidified telemedicine’s pivotal role [19]. Since the onset of the pandemic, observational studies have swiftly unveiled the significant impact of cardiovascular comorbidities on disease progression, underscoring the necessity of limiting hospital visits for these patients to mitigate the risk of contagion [20,21]. While social distancing measures serve as a means to curb viral transmission, they also present challenges for regular follow-up visits, potentially impeding the timely detection of complications or disease progression that may warrant a change in management. Consequently, the primary challenge for HF patients during the pandemic has been to ensure their safety from infection while upholding vigilant monitoring to prevent hospitalizations. It is precisely in this challenging terrain that telemedicine has definitively flourished. Notably, traditional in-person visits have transitioned to video consultations, and patient–provider interactions have shifted to virtual platforms [22]. Although the adoption of telemedicine pathways has posed challenges for both healthcare providers and patients, the experiences documented in the literature have affirmed the efficacy of this approach [23,24,25]. Despite the dramatic repercussions of the pandemic, its evolution has undoubtedly catalyzed this process of innovation, which it is hoped the scientific community can increasingly leverage.

## 3. Telehealth Consultation

The utilization of telehealth consultations (TCs) as a substitute for in-person visits has been significantly accelerated by the coronavirus disease 19 (COVID-19) pandemic. Before the pandemic, the adoption of TCs was hindered by factors such as a lack of familiarity with technology, regulatory and legal concerns, and issues related to reimbursement. However, with many of these barriers disappearing amid the pandemic, TC has rapidly become a viable alternative for patient care [26]. A recent statement by the Heart Failure Society of America (HFSA) has underscored the importance of TC preparation and billing codes to ensure the sustainability of TC programs during the pandemic [27]. Moreover, conducting a follow-up phone call within 14 days of an HF hospitalization has emerged as crucial in reducing readmissions and improving outcomes, thereby supporting TC as a safe and effective alternative for post-discharge follow-up [28,29]. The frequency of TCs can vary and is often determined by professional judgment. Risk stratification tools, such as the Seattle Heart Failure Model [30], could assist in guiding the intensity of telemedicine interventions. Electronic medical record capabilities, such as MyChart^®^ (Epic Systems Corporation, Madison Wisconsin, USA), also support TC by enabling asynchronous medical care in response to non-urgent patient concerns [31].

TC could play an important role in remote patient management, through an integrated healthcare solution that includes telemonitoring and a service platform for real-time interactive patient–clinician communications [32]. The TC intervention has the potential to serve as a helpful tool for HF patients to work together with their clinicians to identify and implement one beneficial modification to their medication plan [33]. It could also evolve into a comprehensive system that involves a variety of healthcare professionals, including a nurse coordinator, cardiologist, psychiatrist, and primary care physician, along with services such as home telemonitoring, patient self-management guidance, and screening and treatment for comorbid depression [34]. TCs were also employed to provide interactive guidance for physical exercises to HF patients [35,36] and to arrange post-discharge remote visits and consultations, enhancing patient support and care management through digital means. The Medly Titrate (Use of Telemonitoring to Facilitate Heart Failure Mediation Titration) [37] study demonstrates that remote patient monitoring and TCs can effectively assist in the titration of guideline-directed medical therapy (GDMT) for HF with reduced ejection fraction. TC significantly increases the number of patients reaching their target medication doses more quickly, without any increase in adverse events [37]. In the TIM-HF2 (Telemedical Interventional Management in Heart Failure II) trial [38], patients with a left ventricular ejection fraction (LVEF) of 45% or lower were randomly assigned to remote patient management (n = 796) or usual care (*n* = 775). The findings from the TIM-HF2 trial indicate that a well-implemented remote patient management program, when applied to a clearly defined heart failure population, has the potential to decrease both the number of days lost to unplanned cardiovascular hospital admissions and all-causes death [38].

Although experiences inclusive of patients primarily with HF with preserved ejection fraction (HFpEF) or HF with mildly reduced ejection fraction (HFmrEF) are scarce, telehealth should not be restricted solely to patients with a left ventricular ejection fraction of <40% [32]. The single-center trial led by Jimenez Marrero et al. demonstrated how a periodic TC program results in improved clinical outcomes and significant cost reduction in a population of chronic HF patients and an LVED of >40% [39].

It is essential to recognize that a successful telemedicine strategy should be tailored to the specific cultural and linguistic characteristics of the patient. As technology continues to play an increasingly prominent role in healthcare, the use of TC as a means of patient–provider interaction is likely to become even more widespread in the future.

## 4. Wearable Technology

Wearable technology includes devices such as patches, clothing, and smartwatches that integrate sensors to monitor various health metrics continuously. These sensors gather data on heart rate, blood pressure, physical activity, fluid levels in the lungs, and irregular heart rhythms [40]. This information is transmitted to platforms that analyze it, aiding in the diagnosis of health conditions, tracking treatment outcomes, and identifying risk factors for health deterioration [41].

Wearable technology has significant implications for patients with HF, a quarter of whom are affected by atrial fibrillation, a condition associated with higher rates of stroke, hospitalizations, and mortality. The Apple Heart Study, which observed over 419,000 participants for eight months, demonstrated that smartwatch-detected irregular pulses corresponded with atrial fibrillation detected by electrocardiogram (ECG) 84% of the time. Although few participants had HF (less than 4%), these findings suggest that wearables could be a valuable tool for monitoring HF patients not already under constant ECG observation or those without CIEDs [42].

The LINK-HF (Multisensor Non-invasive Remote Monitoring for Prediction of Heart Failure Exacerbation) [43] investigated the predictive capabilities of a wearable sensor with multiple functions for HF-related hospital stays. The study included 100 Veterans Affairs Health System patients, with 74% suffering from HF with reduced ejection capacity. The device used was a single-use sensor patch worn on the chest, which monitored ongoing ECG patterns, movement on three axes, skin conductivity, and temperature, along with the user’s activity and position. Data were transmitted in real time to a cloud server through a smartphone. The key parameters measured were heart rate, heart rate variability, frequency of arrhythmias, respiratory rate, general motion, walking patterns, sleep durations, and changes in body angle and posture. A machine learning algorithm was developed to predict hospitalizations for HF. This system showed a sensitivity of 76–88% and a specificity of 87%, with a median advance notice of 6.5 days before hospital admission due to HF [43]. Further research is necessary to confirm whether this technology can enhance patient outcomes.

The remote dielectric sensing (ReDSTM, Netanya, Israel) Food and Drug Administration (FDA)-approved system features two sensors built into a vest [44]. These sensors measure lung fluid levels by detecting shifts in dielectric currents across the center of the right chest area. For data collection, patients wear the vest for just 90 s each day, and the vest is linked to a console. The collected data are then sent to healthcare providers via a specialized web application. The system’s accuracy in assessing lung fluid has been confirmed through comparison with chest computed tomography scans [44]. A preliminary trial with 50 participants indicated that managing patients with ReDSTM guidance led to fewer HF hospital readmissions, in comparison to periods before and after its use [45]. Nevertheless, the SMILE^TM^-HF (Sensible Medical Innovations Lung Fluid Status Monitor Allows Reducing Readmission Rate of Heart Failure Patients) trial—a randomized clinical study aimed at evaluating whether the ReDSTM could help reduce HF readmissions—was halted early by the study sponsor [46]. The application of ReDSTM post-hospital discharge has also been documented in a study that was not randomized [47].

## 5. Cardiac Implantable Electronic Devices

CIEDs such as implantable cardioverter defibrillators (ICDs) and cardiac resynchronization therapy defibrillators (CRT-Ds) provide survival benefits for selected HF patients with reduced ejection fraction (HFrEF) and offer crucial diagnostic information for its management [48]. One key diagnostic marker they offer is thoracic impedance, which reflects the amount of fluid within the chest. Lower thoracic impedance indicates fluid congestion and is inversely proportional to the pressures within the heart chambers [49]. A decrease in thoracic impedance can occur around two weeks before clinical signs of congestion appear, with a predictive sensitivity of 76.9% for HF-related hospital admissions. Moreover, consistently low levels of thoracic impedance are a marker for increased mortality risk [50].

The OptiVol fluid index (Medtronic Inc., Minneapolis, MN, USA) is calculated based on the deviation in measured impedance from a preset standard, with a value exceeding 60 indicating an elevated risk of mortality [51]. Yet, in the OptiLink HF study [52], telemonitoring with OptiVol did not result in a reduction in HF hospitalizations or mortality rates among patients with advanced HF [52]. Eligible participants included those with a recent ICD implant and a history of heart failure hospitalization, diuretic treatment, or increased brain natriuretic peptide levels. They were randomly assigned to receive physician alerts on fluid status via inaudible texts or to continue standard care without alerts. Importantly, medical action followed less than 60% of the alerts where the fluid index exceeded the predefined thresholds. A secondary exploratory analysis demonstrated a notable 39% risk reduction for adverse clinical outcomes when medical interventions were timely taken after fluid alerts, highlighting the importance of integrating telemonitoring with appropriate medical intervention [53].

The CorVue™ algorithm (St. Jude Medical, St. Paul, MN, USA) [54] provides 12 daily thoracic impedance measurements compared to a standardized baseline. Measurements are taken across different vectors, including from the right ventricle (RV) ring to the device case and between the RV coil to the case. However, the CorVue™ system has been criticized for its low sensitivity in the early detection of HF exacerbation [54].

The PARTNERS HF (Program to Access and Review Trending Information and Evaluate Correlation to Symptoms in Patients With Heart Failure) [55] prospective cohort study investigated whether a combined HF diagnostic algorithm could enhance the prediction of HF-related hospital admissions in patients with an ejection fraction (EF) of ≤35% and New York Heart Association (NYHA) class III or IV symptoms who were using a CRT-D [55]. The algorithm was prepared using a separate dataset and would flag a patient as positive if, within a month, they met any two of these conditions: prolonged atrial fibrillation, fast ventricular rate during atrial fibrillation, fluid index above 60, low activity levels, abnormal autonomic indicators (elevated nighttime heart rate or reduced heart rate variability), or significant device therapy responses (insufficient CRT pacing or shocks from an ICD, or a fluid index above 100 alone). Over a year, patients were checked every quarter. A positive result from the algorithm occurred in 43% of the participants (298 out of 694). Those with a positive device diagnosis had a 5.5-fold increase in the likelihood of HF-related hospitalization. The timing of the device checks also affected the algorithm’s accuracy; the risk of hospitalization due to HF at different check frequencies—bi-monthly, monthly, and quarterly—had hazard ratios of 6.6, 5.5, and 3.1, respectively. The algorithm outperformed just using thoracic impedance measurements in forecasting hospital stays due to HF. In summary, monthly assessments of HF device diagnostic data were effective at identifying patients at increased risk of hospitalization in the following month.

The IN-TIME (Implant-based multiparameter telemonitoring of patients with heart failure) [56] study was a randomized trial that assigned 664 patients with NYHA class II or III HF, an EF of 35% or less, and a need for an ICD or CRT-D device, to either telemonitoring with standard care or to standard care alone in a 1:1 ratio [56]. The telemonitoring included daily analysis of CIED data both by local investigators and a central coordination center, ensuring significant events did not go unnoticed. Notable events involved episodes of ventricular and atrial tachyarrhythmias, a low rate of biventricular pacing, an increase in ventricular extrasystoles, reductions in patient activity, and abnormal intracardiac electrograms. The primary outcome was a deterioration in a combined clinical score over 12 months, encompassing both clinical assessments by healthcare providers, like HF hospitalizations, and a global assessment score from the patients’ perspective. The study findings showed that 18.9% of patients in the telemonitoring group experienced a worsening in their clinical composite score versus 27.2% in the standard care group, a significant difference (*p* = 0.013; OR 0.63, 95% CI 0.43–0.90), mainly attributed to increased mortality in the control group. The number of hospitalizations for worsening HF did not differ significantly between the two groups. The authors suggest three mechanisms through which daily monitoring might have improved outcomes: early identification of arrhythmias, early detection of suboptimal device performance (like decreased CRT pacing), and recognition of clinical deterioration through patient calls.

The PARTNERS HF and IN-TIME research indicates that for individuals suffering from symptomatic HFrEF who have CIEDs, a consistent and thorough review of HF diagnostics can assist in identifying exacerbations of HF early on and enhance patient outcomes [55,56].

The HeartLogic HF algorithm (Boston Scientific, Boston, MA, USA) [57] integrates data from various sensors present in commercially available ICD or CRT-D devices. These data include the sounds of the first and third heartbeats, respiratory rate, the rapid shallow breathing index (which is the ratio of respiratory rate to tidal volume), thoracic impedance, heart rate, and physical activity level of the patient. Deviations in the sensor readings from the patient’s established baseline are computed and combined to form the HeartLogic HF index [57]. An alert is issued when this index surpasses a pre-set threshold defined by the user. Patients who receive an alert alongside elevated levels of N-terminal pro–B-type natriuretic peptide (NT-proBNP) have an increased risk, by 50 times, of experiencing an HF event [58]. The average period from when an alert is triggered to the occurrence of an HF event is 34 days, offering a window of opportunity for intervention that could lessen the likelihood of hospitalization [59]. Another valid example has been provided by the SELENE HF (Selection of potential predictors of worsening heart failure) study [60] that permitted the development and validation of an algorithm for the prediction of HF hospitalizations through the combination of remote monitoring data transmitted by ICD and the Seattle HF Model. Devices made by BIOTRONIK (SE & Co. KG, Berlin, Germany), feature home monitoring that automatically sends daily device data over the GSM network, accessible on the webpage for hospital staff. This integrated algorithm demonstrated being able to predict first post-implant HF hospitalizations in two-thirds of selected HF patients [60]. The major randomized controlled trials that compare remote monitoring (RM) to traditional in-office follow-ups for heart failure patients with implanted CIEDs are displayed in Table 1.

## 6. Telemedicine as a Tool for Implementing Medical Therapy: Towards a Paradigm Shift

Despite recommendations emphasizing the absolute importance of reaching target doses of guideline-directed medical therapy (GDMT) for HF, real-world data reveal widespread underutilization of these therapies, with prescriptions often at unjustifiably lower doses compared to those proven effective in clinical trials [67,68].

To achieve proper therapy optimization, telemedicine could represent a paramount resource. Analyzing vital parameters, along with evaluating the clinical picture and recent blood chemistry tests, indeed represents the cornerstones for progress in this direction. 

The impact of this strategy was assessed in the EPIC-HF (Electronically Delivered, Patient-Activation Tool for Intensification of Medications for Chronic Heart Failure with Reduced Ejection Fraction) trial [69]. This study compared usual care, consisting solely of ambulatory visits, with a patient activation tool, which included a 3 min video highlighting the significance of GDMT, among 306 outpatients with HFrEF. At the 30-day mark, patients in the activation arm experienced an almost 20% absolute increase in the initiation or intensification of GDMT. This underscores the substantial impact that enhancing patient knowledge and motivation can exert on GDMT rates [69].

Additionally, multiple studies have supported the effectiveness of a telemedicine pathway based on interviews conducted by nursing or pharmacist staff in facilitating the implementation of pharmacological therapy [68,70,71]. In the systematic review conducted by Yun et al., demonstrating a significant reduction in mortality among patients with HFrEF followed through a telehealth program, the significant implementation and adherence to medical therapy achieved were identified as the primary contributors to the obtained outcome [72].

Therefore, one of the primary goals of telemedicine is to transition the intervention strategy from a ”reactive” approach, where therapy is adjusted in response to worsening symptoms, to a ”proactive” approach, where therapeutic adjustments and titration are made based on changes in monitored parameters during the subclinical phase.

## 7. Remote Invasive Hemodynamic Monitoring

The transition from chronic compensated to acute decompensated HF remains incompletely understood, with mild and underhand symptoms gradually progressing to an HF-related event, often associated with high morbidity and mortality. A pattern of rising cardiac filling pressures forwards HF symptoms and hospitalization by approximately three to four weeks, with a gradual increase, which can potentially be modified by specific therapies [73,74].

Given this pathophysiological background, the ability to identify an elevation in filling pressures would be valuable in preventing progression to an acute HF event, thus reducing its relevant burden.

An initial landmark experience was offered by the COMPASS HF (Chronicle Offers Management to Patients with Advanced Signs and Symptoms of Heart Failure) trial [75]: the use of a continuous hemodynamic monitor led to a nonsignificant reduction in the rate of HF events by 21%. Although the FDA did not approve the device, considering the rate of complications (0.06 events per 6 patient months) and the prolonged initial hospitalizations related to the device (0.02 events per 6 patient months), this study underscored the progressive increase in estimated pulmonary artery diastolic pressure before an acute hospitalization, as well as the value of pulmonary pressure-guided therapies [75].

The CHAMPION (CardioMEMS Heart Sensor Allows Monitoring of Pressure to Improve Outcomes in NYHA Class III Heart Failure Patients trial) trial [76] was later designed to investigate a different kind of sensor, delivered directly in a branch of the pulmonary artery. In the treatment arm, specific pressure targets of systolic (15–35 mmHg), diastolic (8–20 mmHg), and mean (10–25 mmHg) pulmonary pressures were established, prompting adjustments in diuretic agents, neuro-hormonal therapy, long-acting nitrates, and/or patient habits. A significant reduction in the rate of hospitalizations for HF over 6 months was assessed and confirmed in the 17-month follow-up period [76]. The clinical effect and good patient adherence were corroborated across the EF continuum and in a real-world population [77,78].

Following the waves of good results of earlier trials, additional devices have emerged. The LAPTOP (Left Atrial Pressure Monitoring to Optimize Heart Failure Therapy Study) and VECTOR-HF (The V-LAP Left Atrium Monitoring systEm for Patients With Chronic sysTOlic & Diastolic Congestive heart Failure) trials, for instance, have demonstrated promising results in monitoring and targeting left atrial pressures; anyway, the high rates of implantation-related complications necessitated caution [79,80]. An evolution of pulmonary pressure monitoring comes in the form of the Cordella Pulmonary Artery Sensor with its Heart Failure System. The device enhances clinical data collection by providing access to body weight, blood pressure, heart rate, blood oxygen saturation, pulmonary pressure, and symptoms. Consequently, it facilitates a whole patient evaluation, thereby elevating the chances of optimal medical therapy titration [81]. Taken together, a growing body of evidence supports the superiority of hemodynamic-guided HF management in reducing HF hospitalization and mortality [9,10,79,82,83]. Nonetheless, questions regarding the ideal candidate for remote invasive pressure monitoring remain unanswered.

### 7.1. Who Is the Ideal Candidate for Remote Invasive Pressure Monitoring?

In the 2021 European Society of Cardiology (ESC) guidelines on HF, invasive hemodynamic monitoring was suggested for symptomatic patients with HFrEF (EF < 35%) to improve clinical outcomes (class IIb, level of evidence b) [83]. Anyway, no clinical trial identified an EF threshold below which the effect of the devices appeared reduced (Table 2). Both the GUIDE HF (Hemodynamic-GUIDEd management of Heart Failure) and the real-life experiences involving 2000 patients implanted with CardioMEMS (St. Jude Medical, Inc., Saint Paul, MN, USA) have confirmed the efficacy of the devices in the whole HF spectrum [77,78]. These findings led to modified indications in the 2022 America Heart Association (AHA)/American College of Cardiology (ACC) guidelines, which adhere more strictly to the inclusion criteria of the conducted trials (Table 2). RM of pulmonary artery pressure may be considered in selected adult patients with NYHA class III or IV and an HF hospitalization in the previous year or elevated natriuretic peptide levels. This recommendation applies only to those on maximally tolerated stable doses of guideline-directed medical therapy (GDMT) with optimal device therapies (class of recommendation IIb, level of evidence b) [84].

### 7.2. What Is the Role of Remote Invasive Pressure Monitoring in Advanced Heart Failure?

AHF represents a clinical dilemma. This term defines patients who are refractory to traditional therapies and require evaluation for HTx, LVAD, or palliative therapies. The adverse prognostic outlook of this population has led to their exclusion from the main HF trials, with no exception for invasive pressure monitoring (Table 2). Nevertheless, a potential role in remotely monitoring patients listed for HT is emerging. Elevated pulmonary vascular resistance, particularly if refractory to medical therapy, may represent a contraindication to HTx, depending on its severity [85]. No clinical experience regarding using a remote monitor in the “bridge to transplantation” strategy has been described. Yet, clinical trials have demonstrated a net reduction in pulmonary artery pressures with the titration of medical therapies in a population with features similar to the HTx candidates (NYHA class III or IV, recurrent hospitalizations, and high natriuretic peptides values regardless of optimal medical therapy), implying a possible role of these devices in a high-risk population, where monitoring the pulmonary pressure trend could be valuable in identifying the optimal HT “window” [86,87,88]. An intriguing application for these devices could regard patients undergoing periodic or continuous inotropic therapy, where hemodynamic data might provide crucial insights to optimize therapeutic management [89].

**Table 2:** A selection of the main inclusion and exclusion criteria applied in the most relevant trials studying invasive hemodynamic monitoring.

Clinical Trial	Trial Summary	Results	Main Inclusion Criteria	Main Exclusion Criteria
COMPASS [75]	Prospective, multicenter, randomized, single-blind, parallel-controlled trial of 274 patients who received an implantable continuous hemodynamic monitor.	Nonsignificant 21% lower rate of all HF-related events in the treatment arm compared with the control group (*p* < 0.33).	HF patients with NYHA functional class III or IV HF (regardless EF). ACE-I or ARB, and a beta-blocker, as tolerated, for at least 3 months before enrollment. At least 1 HF-related hospitalization or ED visit necessitating intravenous treatment (within the previous 6 months).	PAH. Major CV event within 3 months before enrollment. Severe, noncardiac condition limiting 6-month survival. Serum creatinine higher than 3.5 mg/dL or renal dialysis. Likely to undergo HTx within 6 months of randomization. Receiving continuous inotropic therapy. ICD or CRT in the last 3 months.
CHAMPION [76]	Prospective, multicenter, randomized, single-blind trial of 550 patients who received an implantable hemodynamic monitor (CardioMems)	During the entire follow-up (mean 15 months [SD 7]), the treatment group had a 37% reduction in HF-related hospitalization compared with the control group (158 vs. 254, HR 0.63, 95% CI 0.52–0.77; *p* < 0.0001).	HF patients (symptoms > 3 months) with NYHA functional class III HF regardless of LVEF. At least 1 hospitalization for HF in the last 12 months. In patients with reduced LVEF, a beta-blocker, ACE-I, or ARB should be administrated with stable dosage for at least 1 month.	Implantation of CRT less than 3 months before enrollment. Experienced a major cardiac event (e.g., AMI, stroke) within 2 months of screening visit. eGFR less than 25 mL/min or chronic renal dialysis. Likely to undergo HTx within 6 months of screening visit.
GUIDE-HF [77]	A total of 1000 patients implanted with a PA pressure sensor (CardioMems), randomized 1:1 to a hemodynamically guided management group (treatment) or a control group (control).	Primary events (composite of HF hospitalizations, urgent HF visits, and all-cause mortality at 12 months) lower in the treatment arm (*p* = 0.049); HF hospitalization lower in the treatment group (*p* = 0.0072). Within each EF subgroup, primary endpoint and HF hospitalization rates were lower in the treatment group (HR < 1.0 across the EF spectrum).	NYHA class II-IV HF patients. HF hospitalization in the previous 12 months or elevated BNP levels in the previous 30 days, regardless of left ventricular EF. Stable, optimally titrated GDMT for at least 30 days.	Intolerance to all neurohormonal antagonists. ACC/AHA stage D refractory HF. Received or are likely to receive advanced HF therapy in the next 12 months. NYHA class IV HF patients with continuous or chronic use of scheduled intermittent inotropic therapy for HF and an INTERMACS level of ≤4, or persistence of fluid overload with maximum diuretic intervention. eGFR less than 25 mL/min/1.73 m^2^ and nonresponsive to diuretic therapy or receiving chronic dialysis. Unrepaired severe valvular disease. Implanted with CRT-P or CRT-D for less than 90 d prior to consent.
MEMS-HF [82]	Prospective, non-randomized, open-label, multicenter study with 234 patients to characterize safety and feasibility of using remote PA pressure monitoring in a real-world setting.	HF hospitalizations decreased by 62% (*p* < 0.0001). Mean PAP decreased by 5.1 ± 7.4 mmHg. KCCQ overall/clinical summary scores increased (*p* < 0.0001), as the 9-item Patient Health Questionnaire sum score (*p* < 0.0001).	NYHA class III HF at the time of sensor implantation. Hospitalization for worsening HF within 12 months prior to the system implantation. Patients with reduced LVEF must be receiving stable GDMT, as tolerated.	Candidate for HTx, ventricular device implantation or hospice care in the next 12 months.
LAPTOP [90]	Prospective, multicenter, randomized, un-blinded, controlled clinical trial, assessing the role of a LAP monitoring system.	Enrollment was stopped at 486 due to a perceived excess of procedure-related complications. The annualized HF hospitalization rates for treatment patients were 0.40 versus 0.68 in control patients, RRR 41%, *p* = 0.005.	NYHA class III or ambulatory class IV HF of at least 6 months, regardless of LVEF. At least 1 episode of acute decompensated HF treated with intravenous therapy during the prior year. Maximally tolerated, stable doses of ACEi, ARB, or B-blocker if LVEF is less than 40%.	Intractable class IV HF. Recent ACS, stroke, or left-sided cardiac thrombus. Creatinine more than 2.5 mg/dL. Chronic AF.
VECTOR-HF [91]	Prospective, multicenter, single-arm, clinical trial enrolling 30 patients with HF, implanted with an LAP monitor.	Significant improvements in NYHA class and 6 min walk test distance.	Stage C HF in NYHA functional class III. At least 1 hospitalization for worsening HF within the past year or elevated levels of BNP > 300 pg/mL or NT-proBNP > 1500 pg/mL. Reduced and preserved EF. Optimal medical therapy, including GDMT. Clinically stable for a minimum of 3 months prior to enrolment.	eGFR of <25 mL/min/1.73 m^2^. Untreated severe valvular lesions. LVEDd > 80 mm. PAPs higher than 70 mmHg or PVR higher than 4.0 Wood units.

Abbreviations: ACC: American College of Cardiology; ACE-I: angiotensin-converting enzyme inhibitors; ACS: acute coronary syndrome; AF: atrial fibrillation; AMI: acute myocardial infarction; AHA: American Heart Association; ARB: angiotensin receptor blockers; BNP: brain natriuretic peptide, CV: cardiovascular; CRT-D: cardiac resynchronization therapy—defibrillator; CRT-P: cardiac resynchronization therapy—pacemaker; eGFR: estimated glomerular filtration rate; EF ejection fraction; GDMT: guideline-directed medical therapy; HF: heart failure; HTx: heart transplantation; ICD: implantable cardiac defibrillator; KCCQ: Kansas City Cardiomyopathy Questionnaire; LAP: left atrial pressure; LVEF: left ventricular ejection fraction; LVEDd: left ventricular end-diastolic diameter; NTproBNP: N-terminal pro–B-type natriuretic peptide; NYHA: New York Heart Association; PA: pulmonary artery; PAH: pulmonary arterial hypertension; PAPs: pulmonary artery systolic pressure; PVR: pulmonary vascular resistance.

## 8. Telehealth in Left Ventricular Assist Device and Heart-Transplanted Patients

Strict monitoring is of utmost importance for LVAD patients due to the intricacies of post-implant care, necessitating consistent oversight of multiple parameters. Timely intervention facilitated by continuous remote surveillance could enable the early detection or even prevention of severe and costly complications [92]. Telemonitoring of LVAD patients received a boost during the COVID-19 break and was found to be both safe and feasible as well as useful for managing LVAD care. Most experiences relied on regular phone calls, a 24/7 LVAD emergency line, and an extensive network including home care services. The University of Rochester TeleLVAD Study assessed the feasibility and safety of in-video visits compared to conventional in-person VAD clinic visits for patients who live in remote locations [93]. They successfully managed medication adjustments and LVAD speed change remotely with the help of a home nurse, with high patient satisfaction. Similarly, a German study tested the accessibility, efficiency, and favorable reception of a mobile app that allows for the daily or as-needed transmission of various crucial information such as weight, international normalized ratio, medications, LVAD parameters, symptoms, and photos of the driveline exit [94]. Another method of remotely assessing the status of LVAD patients is by combining hemodynamic feedback from invasive devices such as the CardioMEMS HF system [11,95]. In this particularly complex patient population, the use of invasive hemodynamic monitoring could provide key insights to achieve improved overall management. In the INTELLECT 2-HF, patients with LVAD managed with CardioMEMS showed a significant reduction in pulmonary diastolic pressure, with a net improvement in the 6 min walk distance confirmed by functional and clinical benefits [96]. Additionally, monitoring can be conducted through parameters derived from CIEDs such as ICD and CRT-D. A case report showed a successful home management of worsening HF in an LVAD patient detected by the Boston Scientific (Boston, Massachusetts) HeartLogic device algorithm [57]. Telemedicine also represents a potential strategy for monitoring HTx patients; however, its specific impact on this critically ill group remains uncertain. Amidst the backdrop of the COVID-19 pandemic, there was a worldwide adoption of telehealth services implemented to mitigate the transmission of the virus to at-risk patient groups, such as individuals who have undergone solid organ transplants [97,98]. Single-center experiences involving both adult and pediatric HTx recipients have demonstrated that telemedicine offers a valuable means of maintaining connections with patients and minimizing the necessity for in-person visits, especially during disruptions to routine care [98]. The majority of HTx routine patient visits were shifted to a telemedicine platform, with in-person clinic visits reserved for patients within their first-year post-transplant and for urgent patient visits related to acute illnesses or concerning symptoms. Maintaining adherence to immunosuppression poses a distinctive challenge in the context of HTx, particularly heightened during the challenges imposed by the COVID-19 pandemic. The increased occurrence of patients with immunosuppressive levels outside the target range, as observed in some studies, emphasizes the crucial role of regular home immunosuppressive level monitoring [13]. This aspect should be considered a fundamental component of telemedicine practices for this patient cohort. Additionally, some researchers have directed their attention to the utilization of mobile health (m-Health) to bolster post-transplantation self-management for patients and enhance medication adherence [99,100]. The mHeart study [101] investigated the use of the mHeart mobile application and demonstrated a notable rise in drug adherence compared to standard care, along with a notable improvement in patients’ perceived ease and understanding of medication regimens. Similarly, promising preliminary findings emerged from a study conducted in Taiwan on the development of a mobile app [102]. Another paramount aspect worth mentioning is the RM of graft function. A preliminary prospective study in pediatric heart transplant recipients demonstrated that parents can be successfully trained to conduct echocardiograms at home using a handheld echo device [103]. This method proved feasible and sufficient for the remote qualitative assessment of left ventricular systolic function [104]. The widespread and optimal application of telemedicine in LVAD and heart transplant recipients has not been fully established. Despite these challenges, the prospects for telemedicine in this domain are promising.

## 9. The Role of Artificial Intelligence in Telemedicine: The Future Is Now

Artificial intelligence (AI) is a scientific domain focused on developing models of intelligent behavior and designing computer programs to emulate such behaviors. It excels at performing tasks that necessitate human-like intelligence, grounded on learning principles divided into supervised, unsupervised, and reinforcement learning. Machine learning (ML), the foundation of AI, employs models trained on data to facilitate decision-making and algorithms programmed to tackle specific problems [105]. Deep learning (DL), a subset of ML algorithms, utilizes sophisticated techniques such as neural networks, which are inspired by the human brain’s architecture. This enables computer systems to interpret, construct, and comprehend complex data structures and hierarchies. The integration of AI into diagnostic instruments such as ECG, echocardiography, and angiography, alongside modern procedures such as robotic percutaneous coronary intervention, has played a pivotal role in markedly reducing mortality rates among HF patients. These technological advancements have improved risk assessment and laid the groundwork for tailored individualized medical treatments [106]. While AI is unlikely to replace physicians, it serves as an invaluable tool in augmenting clinical judgment and delivering accurate diagnoses, especially in conditions like HF [107]. In telemonitoring scenarios, patient data are automatically screened, facilitating a structured automated response process. This mechanism initiates direct communication with the patient for minor physiological deviations and, in cases of more severe detected abnormalities, escalates communication to healthcare professionals like nurses or physicians, depending on the gravity of the situation [108]. ML, in conjunction with telemedicine, provides a promising solution for addressing various factors that contribute to the instability of HF, such as reduced coronary perfusion, uncontrolled hypertension, erratic heart rhythms, and non-adherence to medication regimes [109]. This approach enables early therapeutic interventions and encourages behavioral changes to preemptively manage health issues, marking a shift from reactive to proactive treatment strategies based on monitoring parameters during the asymptomatic, subclinical phase [110]. Interestingly, research by Golas et al. revealed that DL methods outshine conventional approaches in predicting 30-day readmissions in HF patients [111]. Furthermore, Kwon et al. demonstrated that a DL-based algorithm was superior in forecasting in-hospital and long-term mortality compared to existing scores like the Get with the Guidelines-Heart Failure Score (GWTG) and the Meta-Analysis Global Group in Heart Failure (MAGGIC) score [112]. The superior performance of DL algorithms is likely due to their capability to process an unlimited array of inputs or features, unrestricted by those only with known associations or theoretically plausible justifications. ML has also shown significant promise in medical research by identifying complex relationships and previously unrecognized subtypes of HF. However, diagnosing HF remains challenging due to its complex nature, as it can stem from both structural and functional cardiac disorders. For instance, leg edema, a symptom often linked to right-sided heart congestion, can also result from various other conditions like chronic venous insufficiency, chronic kidney disease, or medication side effects, leading to potential misdiagnoses [113]. The AI-Clinical Decision Support System (AI-CDSS) stands out as a multi-layered medical assistance platform, consisting of several layers including Data Acquisition and Persistence, Context Recognition and Monitoring, Knowledge Acquisition and Inferencing, Engineering Support, and User Interface Management. Particularly noteworthy is the Knowledge Acquisition and Inferencing Layer, which integrates data-derived rules with expert-generated rules, continuously evolving this knowledge over time [114]. A study by Choi et al. showcased the diagnostic accuracy of AI-CDSS for HF to be 98%, underlining its significant value, particularly in settings where access to HF specialists is limited [115]. Furthermore, initiatives like the one from Montefiore Medical Center, advocating the use of AI in monitoring medication intake through smartphone applications, highlight the potential of real-time monitoring to improve patient adherence, a crucial aspect in managing chronic HF [116]. Overcoming technological barriers is essential for realizing the full potential of telemedicine. This includes the necessity for faster and more stable Internet connections, with 5G technology poised to play a key role in managing the ever-increasing data generation. This evolution in technology not only promises to enhance telemedicine’s effectiveness but also ensures that it becomes an integral part of managing chronic conditions like HF, ultimately leading to improved patient outcomes [117].

## 10. Cost-Effectiveness of Telehealth Interventions in Heart Failure

In an era where telehealth policies are adopted by a quarter of nations globally, assessing the effectiveness and cost-efficiency of telehealth in combating the surge in chronic diseases is crucial. The Whole System Demonstrator program [118], the world’s largest trial on telehealth and telecare, highlighted telehealth’s positive impact on clinical outcomes. However, the economic implications remain partially unclear. Some evidence, particularly in chronic HF management, is promising. Klersy et al. analyzed 21 trials, revealing that telemonitoring for congestive heart failure can be cost saving, largely due to decreased hospital admissions. Their findings suggest cost savings directly correlate with the rate of telehealth implementation [119]. Furthermore, Inglis and team’s systematic review, covering structured telephone support or telemonitoring, confirmed telehealth’s efficacy and cost-effectiveness in managing chronic HF. Among twenty-five studies, the majority showed reduced care costs following telehealth interventions, underlining its potential in cost-effective disease management [120]. In another literature review carried out by Grustam et al., it emerged that many studies lacked a thorough economic analysis of telehealth, often omitting investment costs and only a few studies comprehensively evaluated costs and benefits, generally finding telehealth to be cost-effective, with modest improvements or equal effectiveness to traditional care, despite the typically low methodological quality of these studies [121]. Grustam et al. have proposed another analysis on the cost-effectiveness of telehealth, focusing on the comparison between home telemonitoring (HTM) and nurse telephone support (NTS) against standard care (UC) for chronic heart failure (HF) patients. Their research indicates that both HTM and NTS are practical and financially viable methods to assist chronic HF patients. Specifically, NTS is more cost-effective than UC. Similarly, HTM not only enhances patient survival across all New York Heart Association (NYHA) classes but also proves to be cost-effective when compared to UC [121]. The evidence on telehealth’s economic impacts is scant and unconvincing, necessitating comprehensive studies for reliable conclusions. Moreover, the existing research is methodologically weak, yielding few credible papers. Additionally, accurately assessing telehealth’s effects and cost-effectiveness is challenging due to the lack of quality data and appropriate measures, making it difficult to evaluate specific interventions effectively [122].

## 11. Conclusions and Future Directions

Telemedicine has proven to be a valuable asset in the daily management of HF patients, serving as a versatile resource applicable and impactful across all stages of this complex disease (Figure 1). Enhancing well-structured telemedicine programs within the context of HF represents a dual achievement of both implementation and resource optimization. Tailoring telemonitoring pathways to align with the patient’s clinical history, therapy, compliance, and family support can concurrently improve patient management, thereby positively influencing their outcomes, and mitigate the need for unnecessary outpatient evaluations, hospitalizations, and prolonged hospital stays, particularly in scenarios involving telerehabilitation experience. The possibility to develop personalized programs also represents a significant stride towards precision and personalized medicine. Another important concept related to telehealth is connectivity. Leveraging cutting-edge communication technologies, coupled with growing patient willingness and confidence in using informative communication platforms, can promote effective doctor–patient communication. Moreover, it enables the seamless exchange of clinical information among different healthcare professionals and facilitates the involvement of family members. For future interventions aimed at developing and implementing new remote surveillance models, several key considerations should be considered as follows:

A. Optimize patient selection for telemonitoring programs by identifying the most suitable devices, considering their clinical history, comorbidities, technological capability, and cognitive capacity.
B. Implement an efficient yet user-friendly device and include a short training phase to make device usage accessible even for vulnerable subjects.
C. Expand the use of TC to facilitate medication up-titration and enhance patient adherence to GDMT.
D. romote the adoption of invasive hemodynamic monitoring devices, which have been proven to be safe and effective in reducing hospitalizations and mortality.
E. Enhance the role of telehealth for rehabilitative purposes, as successfully demonstrated in heart transplant recipients.
F. Improve artificial intelligence algorithms to enable multiparametric integration of data collected through available systems, enhancing the accuracy with which HF relapses can be predicted.

## Figures and Tables

**Table 1:** Pivotal RCTs comparing remote monitoring vs. in-office only follow-up in heart failure patients implanted with Cardiovascular Implantable Electronic Devices.

Clinical Trial	Device Type	Sample Size (n)	Primary Endpoint	Comparator	Results
TRUST [61]	ICD	1339	Total in-hospital device evaluation number; Adverse event (deaths, stroke, surgical intervention) rate; Time from arrhythmic event to physician evaluation; Detection of device-related complications.	Conventional ICD follow-up	Telemonitoring in patient care cut in-hospital device checks by 45%, significantly sped up arrhythmic event assessments (*p* < 0.001), and swiftly identified those needing urgent care. It proved as safe as traditional monitoring, showing no safety differences between groups.
CONNECT [62]	ICD, CRTD	1997	Time from clinical event (arrhythmias, CV disease progression, and device issues) to clinical decision.	Standard in-office care	4.6 (RM) vs. 22 (CG) days (*p* < 0.01).
EVOLVO [63]	ICD, CRTD	200	Rate of emergency department or urgent in-office visits for HF, arrhythmias, or ICD-related events.	Remote transmission off	75 vs. 117 visits, 35% reduction (*p* < 0.01).
ECOST [64]	ICD	433	Proportion of patients with ≥1 MAE (deaths and CV/procedure/device-related MAE).	Ambulatory follow-ups	38.5% (RM) vs. 41.5% (CG) (*p* < 0.05 for noninferiority), HR 0.91 (95% CI 0.68–1.23).
IN-TIME [56]	ICD, CRTD	664	Worsened composite score of all-cause death, hospital admission for HF, change in NYHA class, and in patient global self-assessment.	Standard care without telemonitoring for 12 months	18.9% (RM) vs. 27.2% (CG) (*p* = 0.01), OR 0.63 (95% CI 0.43–0.9).
IMPACT [65]	ICD, CRTD	2718	Composite of stroke, systemic embolism, and major bleeding.	Standard follow-up and anticoagulat	2.4 (RM) vs. 2.3 (CG) p100-py, HR 1.06 (95% CI 0.75–1.51).
OptiLink HF [52]	ICD, CRTD	1002	Composite of death and CV hospitalization.	No transmitted alerts	45% (RM) vs. 48.1% (CG), HR 0.87 (95% CI 0.72–1.04).
REMOTE-CIED [66]	ICD	595	Effects of RM on health status; Effects of RM ICD acceptance.	In-clinic group	No effect on KCCQ total score; No effect on FPAS total score.

Abbreviations: CI—confidence interval, CG—control group, CRT-D—cardiac resynchronization therapy defibrillator, CRT-P—cardiac resynchronization therapy pacing (no defibrillator), CV—cardiovascular, FPAS—Florida Patient Acceptance Survey, HR—hazard ratio, ICD—implantable cardioverter defibrillator, KCCQ—Kansas City Cardiomyopathy Questionnaire, MAE—major adverse event, OR—odds ratio, OV—OptiVol (pulmonary congestion) algorithm, PM—pacemaker, ppy—per patient-year, RM—remote monitoring, RR—relative risk.
